# Mindfulness is associated with severity of peripheral neuropathy and related patient-reported outcomes among colorectal cancer patients

**DOI:** 10.1007/s00520-022-07340-8

**Published:** 2022-08-28

**Authors:** Cynthia S. Bonhof, Lonneke V. van de Poll-Franse, Ignace H. de Hingh, Gerard Vreugdenhil, Floortje Mols

**Affiliations:** 1 CoRPS – Center of Research on Psychological Disorders and Somatic Diseases, Department of Medical and Clinical Psychology, Tilburg University, Tilburg, The Netherlands; 2 Department of Research, Netherlands Comprehensive Cancer Organisation (IKNL), Utrecht, The Netherlands; 3 Division of Psychosocial Research and Epidemiology, The Netherlands Cancer Institute, Amsterdam, The Netherlands; 4 Department of Surgery, Catharina Hospital, Eindhoven, Netherlands; 5 Department of Epidemiology, GROW-School for Oncology and Developmental Biology, Maastricht University, Maastricht, The Netherlands; 6 Department of Internal Medicine, Máxima Medical Centre, Eindhoven and Veldhoven, The Netherlands

**Keywords:** Colorectal cancer, Peripheral neuropathy, Mindfulness, Anxiety symptoms, Depressive symptoms, Patient-reported outcomes

## Abstract

**Purpose:**

Despite the detrimental impact of chronic (chemotherapy-induced) peripheral neuropathy PN on patients’ lives, treatment options remain limited. We examined the association between mindfulness and chronic PN symptom severity and impairments in related patient-reported outcomes (PROs) among colorectal cancer (CRC) patients up to 2 years after diagnosis.

**Methods:**

Newly diagnosed stage I–IV CRC patients from four Dutch hospitals were eligible for participation. Patients (*N* = 336) completed a questionnaire on mindfulness (MAAS) at 1 year after diagnosis, and questionnaires on sensory (SPN) and motor peripheral neuropathy (MPN) (EORTC QLQ-CIPN20), anxiety and depressive symptoms (HADS), sleep quality (PSQI), and fatigue (EORTC QLQ-C30) before initial treatment (baseline) and 1 and 2 years after diagnosis.

**Results:**

At 1-year follow-up, 115 patients (34%) and 134 patients (40%), respectively, reported SPN or MPN symptoms. In multivariable regression analyses, higher mindfulness at 1-year follow-up was associated with less severe MPN and fewer anxiety and depressive symptoms, better sleep quality, and less fatigue. Of the patients with SPN or MPN at 1-year follow-up, symptoms had not returned to baseline level at 2-year follow-up in 59 (51%) and 72 (54%) patients, respectively. In this subgroup, higher mindfulness was associated with less severe SPN and fewer anxiety symptoms, depressive symptoms, and fatigue at 2-year follow-up.

**Conclusion:**

Mindfulness was associated with less severe PN and better related PROs among CRC patients with chronic PN. More research is needed to examine the role of mindfulness in the transition from acute to chronic PN.

## Introduction

Peripheral neuropathy (PN) is a common and disabling condition in many cancer patients. In colorectal cancer (CRC), PN is most often caused by the administration of oxaliplatin, primarily leading to sensory peripheral neuropathy (SPN) symptoms (i.e., numbness, tingling, and neuropathic pain in the hands or feet) and motor peripheral neuropathy (MPN) symptoms (i.e., impaired movements, distal weakness, and cramps) [1, 2]. Although chemotherapy-induced PN resolves in the majority of patients after treatment, it becomes a chronic condition in 30% of cancer patients [2]. In addition, PN has been found among cancer patients who did not undergo chemotherapy [3], suggesting that the cancer itself may also be involved in PN development.

PN negatively impacts patient-reported outcomes (PROs), including psychological distress, sleep quality, and fatigue [4–7]. For example, among CRC survivors 2–12 years after diagnosis, we previously showed that survivors with high chemotherapy-induced PN reported more anxiety and depressive symptoms and more fatigue compared with survivors with low chemotherapy-induced PN [5]. In addition, in a study using the same study population as the current study, we found that PN was negatively associated with sleep quality over time [8]. There is thus a large need for effective treatments for (chronic) PN, which are, unfortunately, still limited [9].

In parallel to other chronic pain-related syndromes, psychosocial factors likely play an important role in chronic PN. According to biopsychosocial models of chronic pain, including the well-supported fear-avoidance model, future pain is strongly influenced by the way patients interpret their pain sensations [10, 11]. Maladaptive pain cognitions, such as pain catastrophizing, promote hypervigilance to symptoms and repetitious avoidance of activities, which may result in the persistence and even exacerbation of symptoms. Just as psychological factors can contribute to this negative pain circle [12, 13], some psychological factors, such as mindfulness, may also be able to break through it, and may therefore lead to lower pain severity and less impairments in related PROs [14, 15].

Mindfulness is defined as a non-elaborative, non-judgmental, present-centered awareness in which each thought, feeling, or sensation that arises in the attentional field is acknowledged and accepted as it is [16]. It can be seen as both a dispositional trait that varies from individual to individual, as well as a trainable skill [17]. Among patients with chronic pain, mindfulness is associated with better pain outcomes, such as lower pain severity and less pain interference [18–21], yet little is known about the association between mindfulness and PN. To our knowledge, only one study has been conducted so far. In this study among breast and gastrointestinal cancer survivors with chronic neuropathic pain, higher mindfulness was cross-sectionally associated with lower pain intensity; less pain catastrophizing, pain interference, and depression; and better mental health-related quality of life (HRQoL) [22]. However, it is unclear whether mindfulness is cross-sectionally associated with PN in general (including painful PN) among cancer patients and whether mindfulness is also prospectively associated with PN. Therefore, we will examine the cross-sectional and prospective association between mindfulness and chronic PN symptom severity and related PROs (i.e., psychological distress, sleep quality, and fatigue) in CRC patients. We hypothesize that mindfulness, measured at 1-year follow-up, will be associated with PN symptom severity and related PROs at 1- and 2-year follow-up among our sample of CRC patients.

## Methods

### Setting and participants

This study is based on data from the PROCORE study, a prospective, population-based study aimed to examine the longitudinal impact of CRC and its treatment on patient-reported outcomes. Details of the data collection have been described elsewhere [8]. In short, data was collected through PROFILES, a registry for the physical and psychosocial impact of cancer and its treatment [23]. PROFILES is directly linked to the Netherlands Cancer Registry (NCR), which collects data from all newly diagnosed cancer patients in the Netherlands [24].

Patients were recruited from four Dutch hospitals: Elisabeth-TweeSteden Hospital, Catharina Hospital, Elkerliek Hospital, and Máxima Medical Centre. Eligible patients newly diagnosed with primary CRC between January 2016 and January 2019 were invited. Exclusion criteria were previous diagnosis with cancer, except for basal cell carcinoma of the skin, cognitive impairments, and not being able to read or write Dutch. All patients were included shortly after diagnosis, before start of treatment. In practice, some patients who were previously diagnosed with cancer and those who already started treatment were also included. Parallel to previous PROCORE publications [25, 26], patients were excluded for analysis if they (1) were previously diagnosed with cancer and reported baseline EORTC QLQ-CIPN20 scores > 0 or (2) already started chemotherapy.

### Data collection

Eligible patients were invited by their research nurse or case manager. Patients received an information package, including an information letter, informed consent form, and the first questionnaire. Follow-up questionnaires were sent 4 weeks after surgery (when applicable), and 1 and 2 years after diagnosis. The questionnaire 4 weeks after surgery was not included in the current analyses, as it did not include PN. The PROCORE study was approved by the certified Medical Ethic Committee of Medical Research Ethics Committees United (registration number NL51119.060.14).

### Sociodemographic and clinical characteristics

Patients’ sociodemographic (i.e., age, sex) and clinical (i.e., cancer type, clinical stage, treatment) information was available from the NCR [24]. Questions on partner status and educational level were added to the questionnaire. Comorbidity was assessed with the adapted Self-Administered Comorbidity Questionnaire (SCQ) [27].

### Mindfulness

Dispositional mindfulness was measured at 1-year follow-up using the Mindful Attention Awareness Scale (MAAS) [28]. The MAAS is a validated 15-item scale assessing awareness of and attention to what is occurring in the present, which is a core characteristic of dispositional mindfulness. Items are answered on a 6-point Likert scale ranging from (1) *almost always* to (6) *almost never*. The MAAS is scored by calculating the mean of the items, with higher scores indicating greater dispositional mindfulness.

### Peripheral neuropathy

The EORTC QLQ-CIPN20 [29] was used to assess PN severity, both in patients with and without chemotherapy, as PN has also been found among CRC patients who did not receive chemotherapy [3]. The EORTC QLQ-CIPN20 assesses the extent in which sensory, motor, and autonomic symptoms of PN were experienced during the past week. In the current study, we solely focused on SPN and MPN, and not autonomic PN, as SPN and MPN are most prevalent in CRC, and we previously only found an increase in SPN and MPN after treatment [25]. EORTC QLQ-CIPN20 items are measured on a Likert scale ranging from (1) *not at all* to (4) *very much*. Scores were transformed to a 0–100 scale, with higher scores representing higher symptom severity [30]. The SPN scale was calculated excluding the item on hearing problems [26], as it has been found unlikely to accurately identify PN [31].

### Anxiety and depressive symptoms

The Hospital Anxiety and Depression Scale (HADS) was used to assess anxiety and depressive symptoms [32]. The items, which assess anxiety and depressive symptoms in the last week, are answered on a four-point Likert scale. Total scores for both scales range from 0 to 21, with higher scores representing more anxiety and depressive symptoms.

### Sleep quality

Sleep quality was measured using the Pittsburgh Sleep Quality Index (PSQI) [33]. It consists of 19 items assessing seven components of sleep in the previous month (e.g., subjective sleep quality, sleep latency, sleep duration, and habitual sleep efficiency). A global PSQI score is calculated by the components and ranges from 0 to 21, with higher score representing worse sleep quality.

### Fatigue

The fatigue subscale of the EORTC QLQ-C30 was used to assess fatigue [34]. The fatigue subscale consists of three items, asking patients to what extent they needed to rest, felt weak, and were tired. Items are scored on a Likert scale ranging from (1) *not at all* to (4) *very much*. Scores were linearly transformed to a 0–100 range, with higher scores representing more fatigue [30].

### Statistical analyses

NCR data on patient characteristics enabled us to compare eligible patients and respondents, using *t* tests for continuous variables and chi-squared (or Fisher’s exact) tests for categorical variables. Similarly, differences in patient characteristic were compared between (1) patients with SPN and those without SPN at 1-year follow-up and (2) patients with MPN and those without MPN at 1-year follow-up. Patients were considered to have SPN or MPN if they either developed SPN or MPN symptoms (i.e., EORTC QLQ-CIPN20 SPN/MPN subscale score = 0 at baseline and > 0 at 1-year follow-up) or experienced a worsening of their existing SPN or MPN symptoms at 1-year follow-up (i.e., > 0 difference between EORTC QLQ-CIPN20 SPN/MPN subscale score at baseline and 1-year follow-up) [35].

To provide more insight in the PN symptoms of the patients in this study, frequency distributions were calculated in patients with SPN (or MPN) at 1-year follow-up and patients whose SPN (or MPN) symptoms had not returned to baseline level at 2-year follow-up.

Finally, hierarchical regression analyses were done to examine the association between mindfulness at 1-year follow-up and SPN (or MPN) severity and psychological distress, sleep quality, and fatigue at 1- and 2-year follow-up. In the first step, solely mindfulness was entered in the model. In the second step, a priori-determined sociodemographic (i.e., age, sex, partner status (yes/no), and educational level (high vs. low/medium)) and clinical confounding variables (i.e., tumor type (colon vs. rectum(sigmoid)), cancer stage (stage III/IV vs. I/II), radiotherapy, oxaliplatin, and capecitabine) were added.

All analyses were performed using SPSS (IBM SPSS Statistics for Windows, Version 24.0 Armonk, NY: IBM Corps, USA). A *p* value < 0.05 was considered statistically significant.

## Results

### Patient characteristics

Of the 713 CRC patients who were invited to the study, 68% (*n* = 483) completed the questionnaire at baseline, 52% (*n* = 374) at 1-year follow-up, and 49% (*n* = 347) at 2-year follow-up. A flow chart of the study has been published previously [8]. Compared with all patients eligible for participation, respondents were younger, more often male, and less often diagnosed with rectosigmoid cancer. Furthermore, they were less likely to undergo surgery, more likely to receive chemotherapy, and more often had stage III cancer and less often stage IV cancer (data not shown). While baseline and 1-year follow-up questionnaires were completed by 374 patients, 336 patients were included in the analyses of this study as six patients had missing data on the EORTC QLQ-CIPN20 and 32 patients were previously diagnosed with cancer and reported baseline EORTC QLQ-CIPN20 scores > 0 and/or had already started chemotherapy at time of baseline.

Patients with SPN symptoms at 1-year follow-up (*n* = 115, 34%) were on average younger, less likely to have stage I or II cancer, and more likely to have stage III cancer, and they received chemotherapy and specifically oxaliplatin more often compared with patients without SPN (Table 1). Patients with MPN (*n* = 134, 40%) were more often female, less often had no comorbidities, and more often at least two comorbidities. Furthermore, they more often had osteoarthritis, and more often received chemotherapy, specifically oxaliplatin, compared with patients without MPN.

**Table 1:** Baseline sociodemographic and clinical characteristics of colorectal cancer patients stratified by the presence of sensory and motor peripheral neuropathy at 1-year follow-up

	Sensory peripheral neuropathy		Motor peripheral neuropathy	
	Yes (*N* = 115)	No (*N* = 221)	Yes (*N* = 134)	No (*N* = 202)
Age (mean, SD)	65.1 (9.0)*	67.3 (8.5)	65.3 (9.5)*	67.3 (8.0)
Female sex	44 (38%)	85 (39%)	62 (46%)*	67 (33%)
Partner (yes)	95 (83%)	191 (86%)	111 (83%)	175 (87%)
Education level^a^				
Low	13 (11%)	17 (8%)	11 (8%)	19 (10%)
Medium	67 (58%)	142 (65%)	84 (63%)	125 (62%)
High	35 (30%)	60 (27%)	38 (29%)	57 (28%)
Tumor location				
Colon	85 (74%)	157 (71%)	93 (69%)	149 (74%)
Rectum/rectumsigmoid	30 (26%)	64 (29%)	41 (31%)	53 (26%)
TNM stage				
I	19 (17%)^‡^	87 (39%)	35 (26%)	71 (35%)
II	22 (19%)	71 (32%)	37 (28%)	56 (28%)
III	70 (61%)	56 (25%)6	56 (42%)	70 (35%)
IV	4 (4%)	(3%)	6 (5%)	4 (2%)
Unknown	0 (0%)	1 (1%)	0 (0%)	1 (1%)
Tumor differentiation grade				
Well differentiated	0 (0%)	2 (1%)	0 (0%)	2 (1%)
Moderately differentiated	93 (81%)	178 (81%)	115 (86%)	156 (77%)
Poorly differentiated	6 (5%)	11 (5%)	3 (2%)	14 (7%)
Unknown	16 (14%)	30 (14%)	16 (12%)	30 (15%)
Radiotherapy (yes)	21 (18%)	29 (13%)	18 (13%)	32 (16%)
Chemotherapy				
No	51 (44%)^‡^	181 (82%)	83 (62%)^†^	149 (74%)
Capecitabine	10 (9%)	18 (8%)	9 (7%)	19 (9%)
Oxaliplatin	54 (47%)	22 (10%)	42 (31%)	34 (17%)
Surgery (yes)	112 (97%)	217 (98%)	131 (98%)	198 (98%)
Number of comorbidities				
None	34 (30%)	60 (27%)	33 (25%)	61 (31%)
One	43 (38%)	70 (32%)	47 (35%)	66 (33%)
Two or more	37 (32%)	89 (41%)	54 (40%)	72 (36%)
Comorbidities associated with PN^b^				
Osteoarthritis	26 (23%)	43 (20%)	35 (26%)^†^	34 (17%)
Rheumatoid arthritis	6 (5%)	10 (5%)	7 (5%)	9 (5%)
Diabetes mellitus	13 (11%)	17 (8%)	13 (10%)	17 (9%)

Variables may deviate from 100% due to rounding off. Significant difference between either patients with SPN and those without SPN at 1-year follow-up, or patients with MPN and those without MPN at 1-year follow-up at *p < 0.05; †*p* < 0.01; ‡*p* < 0.001

*SD* standard deviation, *PN* peripheral neuropathy

^a^Education: low (no or primary school); medium (lower general secondary education or vocational training); high (pre-university education, high vocational training, university)

^b^Most frequent comorbidities associated with peripheral neuropathy

### PN

Among patients who reported SPN at 1-year follow-up, tingling fingers or hands (46%), tingling toes or feet (40%), numbness in toes or feet (38%), and numbness in fingers or hands (32%) were most frequently reported. In addition, while among 33% (*n* = 38) of patients SPN symptoms had returned to baseline at 2-year follow-up, 51% (*n* = 59) continued to have SPN levels above baseline (*n* = 18 missing). Specifically, SPN symptoms improved slightly compared to 1-year follow-up, but did not return to baseline in 28% (*n* = 32) of patients, remained stable in 11% (*n* = 13), and worsened in 12% (*n* = 14). Patients reported improvements primarily in tingling toes or feet (37%) and tingling fingers or hands (38%), and deterioration in problems standing or walking because of difficulty feeling the ground under their feet (12%) and numbness in toes or feet (11%).

Among patients with MPN at 1-year follow-up, difficulty opening a jar or bottle because of weakness in hands (62%), difficulty manipulating small objects with fingers (50%), difficulty climbing stairs or getting up out of a chair because of weakness in legs (42%), and cramps in hands (35%) were most frequently reported. At 2-year follow-up, MPN symptoms had returned to baseline in 33% (*n* = 44) of patients, whereas symptoms had not returned to baseline in 54% (*n* = 72, *n* = 18 missing). Specifically, during that year, symptoms had improved somewhat in 22% of patients, remained stable in 16%, and worsened in 16%. Improvements were mainly reported in difficulty opening a jar or bottle because of weakness in hands (25%) and difficulty manipulating small objects with fingers (22%), while deteriorations were mainly reported in cramps in feet (13%) and cramps in hands (10%).

### Mindfulness as a predictor of PN severity and related PROs

At 1-year follow-up, mindfulness was not significantly associated with SPN symptom severity, while higher mindfulness was associated with less severe MPN after controlling for sociodemographic and clinical confounders (Table 2). Mindfulness explained 3% of the variance in MPN symptom severity. Higher mindfulness was also associated with fewer anxiety and depressive symptoms, better sleep quality, and less fatigue among patients with SPN or MPN. Among patients with SPN or MPN, the variance explained by mindfulness was lowest for sleep quality (5% and 6%, respectively) and highest for anxiety symptoms (41% and 47%, respectively).

**Table 2:** Association between mindfulness and PN symptom severity and related PROs among patients with (A) SPN and (B) MPN at 1-year follow-up

	PN^a^ severity		Anxiety symptoms		Depressive symptoms		Sleep quality		Fatigue	
	*β*	Adj *R*^2^	*β*	Adj *R*^2^	*β*	Adj *R*^2^	*β*	Adj *R*^2^	*β*	Adj *R*^2^
(A) Sensory peripheral neuropathy										
*Step 1*										
Mindfulness	− .11		− .67^‡^		− .56^‡^		− .27^†^		− .37^‡^	
		.00		.44^b^		.30^b^		.06^b^		.13^b^
*Step 2*										
Mindfulness	− .15		− .66^‡^		− .54^‡^		− .22*		− .37^‡^	
Age	− .17		.03		.02		− .20*		− .16	
Sex (female)	− .04		.16*		.04		.23*		.04	
Partner (yes)	.06		.12		.11		.19		.13	
Educational level (high)	− .22*		− .14		− .11		− .26^†^		− .15	
Tumor type (colon)	.29*		− .04		− .08		− .02		.03	
Stage (III and IV)	− .15		.04		− .10		− .11		− .14	
Radiotherapy	− .21		− .09		.15		.14		− .01	
Chemotherapy—oxaliplatin	.59^‡^		− .08		− .09		.02		.14	
Chemotherapy—capecitabine	.12		.10		− .02		.00		.20	
		.25^b^		.48^b^		.31^b^		.17^b^		.16^b^
(B) Motor peripheral neuropathy										
*Step 1*										
Mindfulness	− .16		− .72^‡^		− .56^‡^		− .28^†^		− .37^‡^	
		.02		.51^b^		.31^b^		.07^b^		.13^b^
*Step 2*										
Mindfulness	− .18*		− .70^‡^		− .57^‡^		− .26^†^		− .37^‡^	
Age	.04		− .03		− .06		− .19*		− .15	
Sex (female)	.05		.07		− .21*		.05		− .14	
Partner (yes)	.11		.06		.01		.15		.06	
Educational level (high)	− .06		− .14*		− .18*		− .19		− .09	
Tumor type (colon)	− .04		− .02		− .12		− .10		− .13	
Stage (III and IV)	− .13		− .08		− .11		− .09		.05	
Radiotherapy	.02		− .04		.11		.24		− .01	
Chemotherapy—oxaliplatin	.39^†^		− .02		− .12		.03		− .10	
Chemotherapy—capecitabine	.16		− .03		.02		− .21		.20*	
		.07^b^		.52^b^		.36^b^		.11^b^		.17^b^

*PN* peripheral neuropathy, *PROs* patient-reported outcomes, *SPN* sensory peripheral neuropathy, *MPN* motor peripheral neuropathy, *Adj* adjusted

^*^Significant at *p* < 0.05

^†^Significant at *p* < 0.01

^‡^Significant at *p* < 0.001

^a^Among patients with SPN (A), the dependent variable is the sensory scale of the EORTC QLQ-CIPN20 (without the item on hearing problems), while among patients with MPN (B), the dependent variable is the motor scale of the EORTC QLQ-CIPN20

^b^Change in adjusted *R*^2^ is significant

Next, we examined whether mindfulness at 1-year follow-up was prospectively associated with PN symptom severity and related PROs at 2-year follow-up (Table 3). After controlling for sociodemographic and clinical confounders, mindfulness was significantly associated with SPN symptom severity, but not with MPN symptom severity. Mindfulness explained 8% of the variance in SPN symptom severity. Higher mindfulness was also associated with fewer anxiety and depressive symptoms and less fatigue, but no longer with sleep quality in patients whose SPN or MPN symptoms did not return to baseline level. Among patients with SPN or MPN, the variance explained by mindfulness was lowest for fatigue (20% and 15%, respectively) and highest for anxiety symptoms (45% and 33%, respectively).

**Table 3:** Prospective association between mindfulness at 1-year follow-up and PN symptom severity and related PROs at 2-year follow-up among patients with (A) SPN and (B) MPN

	PN^a^ severity		Anxiety symptoms		Depressive symptoms		Sleep quality		Fatigue	
	*β*	Adj *R*^2^	*β*	Adj *R*^2^	*β*	Adj *R*^2^	*β*	Adj *R*^2^	*β*	Adj *R*^2^
(A) Sensory peripheral neuropathy										
*Step 1*										
Mindfulness	− .24		− .68^‡^		− .49^‡^		− .20		− .43^†^	
		.04		.45^b^		.22		.02		.17^b^
*Step 2*										
Mindfulness	− .30*		− .73^‡^		− .51^‡^		− .13		− .49^†^	
Age	− .25		− .09		− .10		− .39^†^		− .18	
Sex (female)	.03		.07		.08		.11		− .08	
Partner (yes)	.18		.23		.25		.03		.13	
Educational level (high)	− .14		.06		.04		− .18		− .07	
Tumor type (colon)	.28		− .06		.02		− .10		− .14	
Stage (III and IV)	− .14		.16		.01		− .47*		− .10	
Radiotherapy	.01		− .22		.08		.14		.19	
Chemotherapy—oxaliplatin	.36		− .09		− .07		.28		.16	
Chemotherapy–capecitabine	.07		.06		− .03		.09		.16	
		.09		.47^b^		.17^b^		.20^b^		.17^b^
(B) Motor peripheral neuropathy										
*Step 1*										
Mindfulness	− .20		− .63^‡^		− .45^‡^		− .25*		− .40^†^	
		.03		.39^b^		.19^b^		.05^b^		.15^b^
*Step 2*										
Mindfulness	− .22		− .61^‡^		− .41^†^		− .23		− .41^†^	
Age	.04		− .09		− .13		− .28*		− .21	
Sex (female)	− .14		− .01		− .20		− .24		− .36^†^	
Partner (yes)	.01		− .01		− .11		.08		− .06	
Educational level (high)	.12		.01		.16		− .08		.05	
Tumor type (colon)	− .20		− .08		− .19		− .20		− .27	
Stage (III and IV)	− .17		− .04		.00		− .27		− .17	
Radiotherapy	.08		− .13		.06		.12		.29	
Chemotherapy—oxaliplatin	.20		.14		− .22		.14		.00	
Chemotherapy—capecitabine	.22		− .02		− .05		.02		.06	
		.01		.37^b^		.16^b^		.10		.23^b^

*PN* peripheral neuropathy, *PROs* patient-reported outcomes, *SPN* sensory peripheral neuropathy, *MPN* motor peripheral neuropathy, *Adj* adjusted

^*^Significant at *p* < 0.05

^†^Significant at *p* < 0.01

^‡^Significant at *p* < 0.001

^a^Among patients with SPN (A), the dependent variable is the sensory scale of the EORTC QLQ-CIPN20 (without the item on hearing problems), while among patients with MPN (B), the dependent variable is the motor scale of the EORTC QLQ-CIPN20

^b^Change in adjusted *R*^2^ is significant

## Discussion

In this prospective, population-based study among CRC patients, we examined the cross-sectional and prospective association between mindfulness and chronic PN symptom severity and impairments in related PROs. We first showed that 34% of patients developed SPN or experienced a worsening of their existing SPN at 1-year follow-up, of whom 51% still reported SPN levels above baseline level at 2-year follow-up. Regarding MPN, development or worsening of existing symptoms was reported by 40% of patients, of whom 54% still reported MPN levels above baseline level at 2-year follow-up. Interestingly, 12–16% of patients reported worsening of symptoms at 2-year follow-up compared with 1-year follow-up. While coasting—the phenomenon that CIPN symptoms continue to worsen for 3 months after discontinuation of chemotherapy—has been found among patients treated with oxaliplatin [36], our patients had already completed their chemotherapy for ≥ 4 months at 1-year follow-up. Therefore, the worsening of symptoms at 2-year follow-up is likely related to older age and age- and PN-related comorbidities (e.g., rheumatoid arthritis, diabetes mellitus). Since we only have data on chemotherapy as primary treatment, it is also unknown whether patients may have undergone additional chemotherapy (e.g., for recurrent disease), which could exacerbate existing PN symptoms.

We hypothesized that mindfulness, measured at 1-year follow-up, would be associated with PN symptom severity and related PROs at 1- and 2-year follow-up. Results showed that higher mindfulness at 1-year follow-up was cross-sectionally associated with less severe MPN and fewer anxiety and depressive symptoms, better sleep quality, and less fatigue, and prospectively with less severe SPN and less anxiety symptoms, depressive symptoms, and fatigue. Only one previous study examined the association between mindfulness and PN. While this study focused on neuropathic pain, results were in line with our findings; mindfulness was negatively correlated with pain intensity and depression, and positively correlated with mental HRQoL [22]. Our findings regarding the association between mindfulness and PN symptom severity were not completely in line with our hypotheses: mindfulness was only associated with MPN symptom severity at 1-year follow-up and with SPN symptom severity at 2-year follow-up. We find it difficult to offer a sensible explanation for this surprising finding. It has previously been hypothesized that chemotherapy-induced PN during chemotherapy is primarily caused by the neurotoxic effects of the chemotherapeutic agent, while chronic chemotherapy-induced PN is primarily influenced by psychological factors that maintain chemotherapy-induced PN symptoms [37], and on which mindfulness may exerts its effect [15]. At 1-year follow-up, PN may not have been chronic for all patients, as some patients only completed their chemotherapy 4 or 5 months earlier. This may explain why we only found an association between mindfulness and SPN symptom severity at 2-year follow-up and not at 1-year follow-up. The finding that oxaliplatin was not related to SPN symptom severity at 2-year follow-up is also in line with the beforementioned hypothesis on the role of psychological versus neurotoxic factors in chronic PN. However, it could also be an indication of good patient care. Patients may have been closely monitored for early signs of PN and were given dose reduction or changes in chemotherapy doses or regimen could have been made when PN occurred, reducing the chance of long-lasting PN [2, 38]. In contrast to the hypothesis of psychological factors on chronic PN, mindfulness was only associated with MPN symptom severity at 1-year follow-up. However, weakness in hands and weakness in legs were among the top MPN symptoms reported at 1-year follow-up. At that time, these symptoms may still be a direct consequence of the cancer and its treatment, rather than reflect actual MPN. This could explain why we found an association between mindfulness and MPN symptom severity at 1-year follow-up, but not at 2-year follow-up. More research is needed to examine the role of psychological factors—including mindfulness—versus neurotoxic factors, both in acute and chronic PN. Nevertheless, while mindfulness was not associated with MPN symptom severity at 2-year follow-up, it was still associated with better related PROs among patients with MPN.

The association between mindfulness and PN symptom severity and related PROs may be explained through catastrophizing. Catastrophizing can be characterized by the perceived lack of control over (pain) symptoms, rumination, magnification of their consequences, and the expectation of negative outcomes [22]. It is a key variable in the fear-avoidance model [10, 11] and has been found to account for 7–31% of the variance in pain severity [39]. This maladaptive coping strategy promotes hypervigilance and avoidance of activities, causing increased distress and functional disability, which eventually leads to further future pain and impairments in related PROs. Mindfulness may interrupt this fear-avoidance cycle through reducing catastrophizing [15, 22]. In the study among cancer survivors with chronic neuropathic pain [22], mindfulness acted as a moderator in the association between pain intensity and pain catastrophizing. That is, among cancer survivors with high levels of pain, mindfulness counteracted patients’ tendency to catastrophize. This may then make patients with high mindfulness less likely to avoid activities they expect to cause pain—or PN symptoms—which lowers their risk of developing (further) emotional distress and functional disability. Indeed, in the previously mentioned study among cancer survivors with neuropathic pain [22], mindfulness also moderated the association between pain intensity and the interference of pain.

Causality cannot be determined in our study, and, to our knowledge, the effectiveness of a mindfulness-based intervention in reducing PN or improving PROs has not yet been examined among cancer patients with (chronic) PN. In other chronic pain-related conditions, mindfulness-based interventions have been shown promising [18, 40, 41]. For example, among patients with painful diabetic peripheral neuropathy whose pharmacotherapy had been optimized, treatment with mindfulness-based stress reduction resulted in reduced pain intensity, pain catastrophizing, depression, and perceived stress, and better HRQoL compared with usual care [40]. Future research is needed to examine the effectiveness of such mindfulness-based interventions in chronic PN.

Several limitations should be mentioned. First, while important determinants of PN symptom severity, we had no data on chemotherapy dosage, number of cycles, and dose reductions [2]. Also, we only used the patient-reported EORTC QLQ-CIPN20 to assess PN, whereas it has been advised to combine self-reported PN measures with clinician-rated neurological assessment tools [42]. Nevertheless, we think that a patient-reported measure of PN is more reliable, as healthcare professionals often underestimate PN severity and objective measurements are often too insensitive to detect beginning or mild PN [43, 44]. The EORTC QLQ-CIPN20 is also the most implemented patient-reported measure of PN, with good psychometric properties, and we adhered to recent guidelines that advised to take caution with the use of its hearing problems item [31]. Another limitation is that we do not know why some patients stopped participating in our study. A previous study found that participants who were lost to follow-up in the PROFILES registry reported significantly worse HRQoL, functioning, and psychosocial symptoms [45]. For example, in our study, patients could have stopped participating because of severe PN symptoms in hands, which could have led to an underestimation of PN prevalence. Finally, generalization of the findings of this study should be done with caution, as eligible patients and participants differed in some sociodemographic and clinical characteristics. Despite these limitations, this study provides important insights in the role of mindfulness in chronic PN and impairments in related PROs. To the best of our knowledge, this is the first study on the role of mindfulness in chronic PN, including both painful and non-painful symptoms, and that examined both the cross-sectional and prospective association between mindfulness and chronic PN and impairments in related PROs. Future, longitudinal studies are warranted that also examine the role of mindfulness in the transition from acute to chronic PN.

In conclusion, the results of this prospective, population-based study among CRC patients showed that higher mindfulness at 1-year follow-up was associated with less severe PN, anxiety, and depressive symptoms, better sleep quality, and less fatigue. Future studies are needed that examine the role of mindfulness in the transition from acute to chronic PN.

## Data Availability

The data that support the findings of this study are available from the PROFILES Registry (www.profilesregistry.nl).

## Abbreviations

ACT: Acceptance and commitment therapy (ACT)
CRC: Colorectal cancer
MPN: Motor peripheral neuropathy
NCR: Netherlands Cancer Registry
PROs: Patient-reported outcomes
PROFILES: Patient-Reported Outcomes Following Initial Treatment and Long-Term Evaluation of Survivorship
PN: Peripheral neuropathy
SPN: Sensory peripheral neuropathy
